# Development of a Multilocus Sequence Typing (MLST) scheme for *Treponema pallidum* subsp. *pertenue*: Application to yaws in Lihir Island, Papua New Guinea

**DOI:** 10.1371/journal.pntd.0006113

**Published:** 2017-12-27

**Authors:** Charmie Godornes, Lorenzo Giacani, Alyssa E. Barry, Oriol Mitja, Sheila A. Lukehart

**Affiliations:** 1 Department of Medicine, University of Washington, Seattle, Washington, United States of America; 2 Department of Global Health, University of Washington, Seattle, Washington, United States of America; 3 Division of Population Health and Immunity, Walter and Eliza Hall Institute, Parkville, Australia; 4 Department of Medical Biology, University of Melbourne, Parkville, Australia; 5 Barcelona Institute for Global Health, Hospital Clinic-Universitat de Barcelona, Barcelona, Spain; 6 Division of Public Health, School of Medicine and Health Sciences, University of Papua New Guinea, Port Moresby, Papua New Guinea; 7 Lihir Medical Center, International SOS-Newcrest Mining, Lihir Island, Papua New Guinea; McGovern Medical School at UTHealth, UNITED STATES

## Abstract

**Background:**

Yaws is a neglected tropical disease, caused by *Treponema pallidum* subsp. *pertenue*. The disease causes chronic lesions, primarily in young children living in remote villages in tropical climates. As part of a global yaws eradication campaign initiated by the World Health Organization, we sought to develop and evaluate a molecular typing method to distinguish different strains of *T*. *pallidum* subsp. *pertenue* for disease control and epidemiological purposes.

**Methods and principal findings:**

Published genome sequences of strains of *T*. *pallidum* subsp. *pertenue* and *pallidum* were compared to identify polymorphic genetic loci among the strains. DNA from a number of existing historical *Treponema* isolates, as well as a subset of samples from yaws patients collected in Lihir Island, Papua New Guinea, were analyzed using these targets. From these data, three genes (*tp0548*, *tp0136* and *tp0326)* were ultimately selected to give a high discriminating capability among the *T*. *pallidum* subsp. *pertenue* samples tested. Intragenic regions of these three target genes were then selected to enhance the discriminating capability of the typing scheme using short readily amplifiable loci. This 3-gene multilocus sequence typing (MLST) method was applied to existing historical human yaws strains, the Fribourg-Blanc simian isolate, and DNA from 194 lesion swabs from yaws patients on Lihir Island, Papua New Guinea. Among all samples tested, fourteen molecular types were identified, seven of which were found in patient samples and seven among historical isolates or DNA. Three types (JG8, TD6, and SE7) were predominant on Lihir Island.

**Conclusions:**

This MLST approach allows molecular typing and differentiation of yaws strains. This method could be a useful tool to complement epidemiological studies in regions where *T*. *pallidum* subsp. *pertenue* is prevalent with the overall goals of improving our understanding of yaws transmission dynamics and helping the yaws eradication campaign to succeed.

## Author summary

Yaws is a neglected treponemal infection that is often transmitted among children in developing countries. Eradication programs in the 1940–50’s significantly reduced the incidence of yaws, but the disease has resurged. The World Health Organization has proposed to eliminate yaws by 2020, and mass treatment trials are underway in a number of countries. To assist in investigating the molecular epidemiology of yaws, we propose a new method for differentiating strains of the causative agent, *Treponema pallidum* subsp. *pertenue*. Using this typing method, we identified seven molecular types in yaws patients from a small island in Papua New Guinea. This method may prove useful in clarifying reinfection vs. relapse, in detecting cases newly imported into a village, in tracking the development of macrolide resistant strains, and in helping to define the transmission of yaws strains within a region.

## Introduction

Yaws is a highly contagious treponemal infection caused by the bacterium *Treponema pallidum* subsp. *pertenue* (*T*.*p*. *pertenue*). It is transmitted by direct skin contact and is symptomatic predominantly in children <15 years of age, usually manifesting as chronic ulcers on the extremities. Latent, or inapparent, infection can persist for decades, often re-emerging as skin lesions or causing painful bone and joint damage [1,2]. Yaws continues to be endemic in a number of tropical countries, particularly in rural regions with lack of public health surveillance. In 2012, the World Health Organization (WHO) proposed a program to eradicate yaws by 2020 [3] using mass drug administration (MDA) with single dose azithromycin. To aid in post-MDA surveillance, a molecular typing scheme is needed to discriminate among *T*.*p*. *pertenue* strains, thus permitting investigators to track the movement of genetically distinct strains in populations and to identify strains newly introduced to already-treated populations. Careful molecular epidemiological studies using typing can assist in understanding the dynamics of disease transmission to improve control of future outbreaks.

*T*.*p*. *pertenue* is closely related to *T*. *pallidum* subsp. *pallidum*, the causative agent of venereal syphilis, which differs from *pertenue* by less than 0.2% of their genome sequences [4]. These subspecies are indistinguishable serologically and morphologically [1,2], but can be differentiated on the basis of molecular signatures [4–9]. For a number of years, molecular typing has been used worldwide for typing treponemes from syphilis patients. This method is based upon 1) the number of 60-base pair repeats in the acidic repeat gene (*arp)* gene (*tp0433*); 2) the restriction fragment length pattern of the Subfamily II *Treponema pallidum* repeat (*tpr*) *E*, *G*, and *J* genes (*tp0313*, *tp0317*, and *tp0621*, respectively) [10], and 3) is enhanced by inclusion of the sequence of a polymorphic 300 bp region of the *tp0548* gene [11]. This typing scheme has been adopted globally in recent years to create a molecular epidemiology database for syphilis, and also to analyze linkage of specific *T*.*p*. *pallidum* molecular types to specific disease manifestations [11,12]. Nonetheless, the 1) well-recognized difficulty in amplifying the *arp* and *tprE/G/J* loci in samples where treponemal DNA is not abundant, 2) the concerns that amplification of the *arp* target might yield inconsistent results [13,14], and 3) the difficulty that sometimes arises in identifying unambiguously the *tprE/G/J* restriction patterns have prompted investigators to propose modifications to the typing approach. These include multilocus sequence typing (MLST) approaches with the capability of discriminating genetic differences among syphilis strains without the risk of ambiguous results. New target loci have included *tp0136* [5,8,15–17] and *tp0279* [18]. Compared to typing methods that rely on restriction fragment length polymorphisms or analysis of tandem repeats, a MLST of proven efficacy would also be more likely to be routinely adopted in research and clinical laboratories.

To provide a better understanding of the current yaws status and to guide control efforts, development of a molecular typing method for *T*.*p*. *pertenue* is highly desirable. Therefore, we sought a sequenced-based typing method using small gene regions that can readily be amplified even from clinical samples with low concentrations of *T*. *pallidum* DNA and whose analysis could unambiguously identify yaws isolates carrying different genetic signatures in these loci.

We propose a MLST method for differentiating *T*.*p*. *pertenue* strains using defined regions of *tp0548*, *tp0136* and *tp0326*. Each of these genes codes for putative (*tp0548*) or bona fide (*tp0136* and *tp0326*) treponemal surface-exposed proteins shown to be implicated in maintaining the homeostasis of the bacterial cell envelope (*tp0326*) [19,20], in mediating adhesion to host components (*tp0136*) [8,21], or hypothesized to mediate nutrient acquisition (*tp0548*). These typing targets yield a highly discriminating molecular method for distinguishing *T*.*p*. *pertenue* strains.

## Methods

### Sources of *T*. *pallidum* subsp. *pertenue* strains

Historical *T*.*p*. *pertenue* isolates (Table 1) were propagated in New Zealand white rabbits by intratesticular inoculation as previously described [22]. DNA was extracted for PCR amplification using the QIAamp DNA Mini Kit (Qiagen, Valencia CA) following the manufacturer’s instructions, but adding 50 μl of proteinase K (100 mg/ml stock solution) instead of 20 μl and incubating the sample for 2 hours at 56°C. Samples were eluted in 200 μl of H_2_O and stored at -20C until used for PCR.

**Table 1:** *Treponema pallidum* subsp. *pertenue* strains used in this study.

	Strain name	Source	Location	Year of isolation	Ref.
Human isolates of *T*.*p*. *pertenue*	Gauthier ^a^	Skin lesion	Nigeria	1963	[23]
	CDC1^b^	Skin lesion	Densuso, Ghana	1980	[24]
	CDC2^b^	Skin lesion	Akorabo, Ghana	1980	[24]
	Samoa D ^c^	Skin	Western Samoa	1953	[25]
	Samoa F^c^	Skin	Western Samoa	1953	[25]
	Ghana051^d^	Unknown	Ghana	1988	[26]
Simian isolate	Fribourg-Blanc ^c^	Lymph node	Guinea	1966	[27]

^a^ Provided by Peter Perine, Centers for Diseases Control and Prevention, Atlanta, GA.

^b^ Provided by Robert George and Victoria Pope, Centers for Disease Control and Prevention, Atlanta, GA.

^c^ Provided by Paul Hardy and Ellen Nell, Johns Hopkins University, Baltimore, MD.

^d^ Provided by Leo Schouls, National Institute for Public Health and Environment, Bilthoven, and Gerda Noordhoek, Public Health Laboratory, Friesland, The Netherlands. DNA only, no known isolate in existence.

### Patient samples

Swab samples containing *T*.*p*. *pertenue* were collected from study participants with exudative skin ulcers in Lihir Island, Papua New Guinea (PNG), during a yaws elimination campaign, between May 2013 and October 2016. Following baseline examination and sample collection, mass treatment with single dose azithromycin was administered. Treatment coverage was 84%. The population was re-examined at 6 month intervals for 42 months. At re-examination, swabs were collected from individuals with yaws-like ulcers, and targeted azithromycin treatment was provided to these persons and their family/childhood contacts. Details of the study have been published elsewhere [28,29]. Immediately after collection, the swabs were placed in 1 ml of 1x lysis buffer (10mM Tris-HCl, 0.1mM EDTA, 0.5% SDS), frozen, and transported to the University of Washington. DNA was extracted using the QIAamp DNA Mini Kit (Qiagen) according to the manufacturer’s instructions. Presence of *T*. *pallidum* DNA was assessed initially by PCR of the Tp47 gene (*tp0574*), which is conserved in all Treponema subspecies, including *pertenue*. Samples positive for *T*. *pallidum* DNA underwent amplification of the *tp1031* (*tprL*) gene as previously described [30] and the amplicon size was used to determine the *pertenue* (vs. *pallidum*) subspecies. All *T*. *pallidum-positive* samples from Lihir Island (n = 232 with duplicates removed), corresponding to 30.7% of all lesion samples analyzed) were confirmed as *T*.*p*. *pertenue* and were used for molecular typing studies. Of these samples, 83.6% (n = 194) were fully typeable with our approach.

### Ethics statement

Participants with suspected yaws or, for young children, their parents or guardians provided written consent for inclusion in clinical surveys and etiological studies, including collection of swabs used in this study. The study was approved by the National Medical Research Advisory Committee of the Papua New Guinea Ministry of Health (MRAC no. 12.36). Only coded samples were sent to the University of Washington for testing.

### Evaluation of typing targets, amplification, sequencing, and sequence analysis

Based upon published *T*.*p*. *pallidum* and *pertenue* genome sequences, we evaluated a number of genes that are polymorphic among strains; these included *tp0136*, *tp0548*, *bamA* (*tp0326*), *tprC* (*tp0117*), *tprD* (*tp0131*), and *tp0619*. Published full length sequences of these genes were initially examined from three human *T*.*p*. *pertenue* strains (Gauthier, CDC2, Samoa D; Table 1) and the Fribourg-Blanc simian isolate. The members of the *tpr* gene family (*tprC* and *tprD*) were not examined further due to the high homology between these two genes and other members of the *tpr* family, making specific amplification problematic. With the exception of *tp0548*, which has an already-identified region that is used for *T*. *pallidum* subsp. *pallidum* typing, we identified, within each of the remaining targets (*tp0136*, *tp0326*, and *tp0619*), regions containing polymorphisms potentially suitable to differentiate the strains. Primers were designed to amplify large regions of these genes for preliminary sequence analysis (Table 2). From 95 *T*.*p*. *pertenue*-positive PNG samples collected from baseline through 18 months of the study, we were able to successfully amplify and obtain good sequences from 66 (69%) samples for *tp0619*, 87 (92%) for *tp0136*, and 42 (44%) for *tp0326*. PCR amplifications for these targets were performed using genomic DNA in a 50-μl final volume containing 200 μM deoxynucleoside triphosphates, 1.5 mM MgCl_2_, 0.8 μM primers (Table 2) and 2.5 U of GoTaq DNA polymerase (Promega, Madison, WI). Cycling conditions for the *tp0136* PCR were 95°C for 3 mins, then 45 cycles of 95°C for 1 min, 60°C for 2 min, 72°for 1 min; followed by 72°C for 10 mins. The conditions for *tp0326* were 95°C for 3 mins, then 45 cycles of 95°C for 1 min, 56°C for 1 min, 72°for 1 min; followed by 72°C for 10 mins. For *tp0619*, cycling conditions were 95°C for 5 mins, then 45 cycles of 95°C for 1 min, 55°C for 1 min, 72°for 1 min; followed by 72°C for 10 mins.

**Table 2:** Primers used for the preliminary analysis of a subset of PNG samples.

Gene	Primer	Sequence 5’-3’	Product Size (nt)	ORF Region (nt)
*tp0619*	Sense	5’-TACAAGCTCCCACAATGCCA-3’		
	Antisense	5’-TTACCCAGACATTTTCTCCACATA-3’	653^1^	224–798
	Sequencing	5’-TACAAGCTCCCACAATGCCA-3’		
*tp0136*	Sense	5’-GAAGAGGGCGTTTTGTG-3’		
	Antisense^2^	5’-CTCCCAGCTCAGCCGAATCTC-3’	1670^2^	1–1485
	Sequencing	5’-GAAGAGGGCGTTTTGTG-3’		
		5’-CTCCCAGCTCAGCCGAATCTC-3’		
		5’-CCATCCAGTCGGAAGTGC-3’		
		5’-AACTACGTAGATTTTCTGCAC-3’		
*tp0326*	Sense	5’-CTGACGGTGGGCTTTGAC-3’		
	Antisense^3^	5’-GCATCTATGACGGCAAAGCG-3’	1169^3^	1538–2559
	Sequencing	5’-AGCACGCCGTCTATTACCAG-3’		
		5’-AAGAGCATTCGTTTCGCTCC-3’		
		5’-CTGACGGTGGGCTTTGAC-3’		

^1^ Amplicon also contains *tp0618*-*tp0619* intergenic region (58 nt) and 20 nt of *tp0618*

^2^ Amplicon also contains *tp0135*-*tp0136* intergenic region (20 nt at 5’-end of *tp0136*) and 165 nt of *tp0137*

^3^ Amplicon also contains *148 nt of tp0327*

Based on the alignments from the historical strains and 95 initial PNG samples, *tp0619* proved not to be a suitable typing target: all historical strains had the same *tp0619* sequence and there were 5 types identified in the amplified PNG samples (S1 Fig). In comparison, even from the low number of *tp0326* sequences that we obtained with these initial primers, we were able to identify 8 *tp0326* types. Thus, the three targets selected for further investigation were *tp0548*, *tp0136*, and *tp0326*. Based upon analysis of these large amplicons, we identified relatively short regions containing polymorphisms yielding the maximum number of unique “types” among the samples tested, and selected those as typing targets for our MLST protocol. Primers, amplicon size, and region identifications are shown in Table 3. Amplifications of these targets (*tp0548*, *tp0136*, and *tp0326* gene fragments) were performed using genomic DNA in a 50-μl final volume containing 200 μM deoxynucleoside triphosphates, 1.5 mM MgCl_2_, 0.8 μM primers (Table 3) and 2.5 U of GoTaq DNA polymerase (Promega, Madison, WI). Thermocycling conditions for *tp0548* have previously been described [11]. Conditions for the *tp0136* PCR were 95°C for 3 mins, then 45 cycles of 95°C for 1 min, 59°C for 2 min, 72°for 1 min; followed by 72°C for 10 mins. The conditions for *tp0326* were 95°C for 5 mins, then 45 cycles of 95°C for 1 min, 58°C for 1 min, 72°for 1 min; followed by 72°C for 10 mins. All amplified products were treated with ExoSAP-IT PCR Product Cleanup Reagent (Affymetrix, Santa Clara CA) for dye deoxy terminator sequencing in one direction. If ambiguities in base-calling were seen in the electropherograms, we repeated the sequencing in both directions, repeating the PCR when necessary. Further, all gene alleles described in our study were found in more than one clinical sample, thus providing confidence that the typing sequences are correct.

**Table 3:** Primers used for the MLST scheme.

Gene	Primer	Sequence 5’-3’	Product Size (nt)	ORF Region (nt)
*tp0548*	Sense^1^	5’-GGTCCCTATGATATCGTGTTCG-3’	300	130–212
	Antisense^1^	5’-CGTTTCGGTGTGTGAGTCAT-3’		
	Sequencing	5’-GTCATGGATCTGCGAGTGG-3’		
*tp0136*	Sense	5’-CCATCCAGTCGGAAGTGC-3’		223–675
	Antisense^2^	5’-CATATCGAGAAACTGTTCGCC-3’	563	
	Antisense^3^	5`-CGTGCAGGCAGAACTCATT-3’	464	
	Sequencing	5’-CCATCCAGTCGGAAGTGC-3’		
*tp0326*	Sense	5’-AAGAGCATTCGTTTCGCTCC-3’	441	2031–2342
	Antisense	5’-CCGGACCGTAGCTCATTTTG-3’		
	Sequencing	5’-GACACCAAGGCCGAGTTCTA-3’		

^1^ Published *tp0548* primers [11]

^2^ Antisense primer for *tp0136* subtypes A-F

^3^ Antisense primer for *tp0136* subtype G

Sequence analysis and alignments of the six human yaws strains of *T*. *pallidum* subsp. *pertenue* (Gauthier, Ghana051, CDC1, CDC2, Samoa D, Samoa F), the Fribourg-Blanc strain, and the PNG samples were performed using Bioedit [31] (http://www.mbio.ncsu.edu/BioEdit/bioedit.html) and Clustal W (http://www.ebi.ac.uk/Tools/msa/clustalw2/). Phylogenetic analysis was conducted by first constructing multiple alignments using the Muscle algorithm implemented in Molecular Evolutionary Genetics Analysis (MEGA) version 7.0 software (http://www.megasoftware.net/) [32], and drawing phylogenetic trees using the Neighbor-Joining method and the number of differences model, with pairwise deletion of gaps and 1000 bootstrap repetitions.

GenBank accession numbers for the new *tp0548* types are as follows: R, MF425823; S, MF425824; T, MF425825; U, HM585227; V, HM243495; W HM245777; X, CP020365. The authors that described *tp0548* types M, N, P, and Q did not submit the sequences to GenBank, so no accession numbers are available The *tp0136* types A-G are MF425826-MF425831 and MF425833. The *tp0326* types 1–8 are MF425834-MF425836 and MF425838-MF425842.

## Results

### Definition of the MLST typing targets

Based upon our analyses of historical strains and a subset of PNG clinical samples, we chose *tp0136*, *tp0548*, and *tp0326* as the most promising targets for use as a *T*.*p*. *pertenue* typing system. Primers were designed and tested for amplification of these regions and those with robust amplification were selected for the MLST scheme (Table 3) The targets, all of which are putative or bona fide outer membrane proteins, each contain small (300–600 nt) readily amplifiable regions with sequence heterogeneity among strains. While the selection of additional, or longer, targets could have increased discrimination, we weighed the resulting requirement for increased sample volume, cost, and time of adding more targets with the risk of losing the ability to fully type some samples. The proposed nomenclature for different *T*.*p*. *pertenue* strain types is expressed as two letters, representing *tp0548* and *tp0136* types, followed by a number, representing *tp0326* types, e.g. JG8.

Using these new MLST primers, we attempted to amplify and sequence typing products from 232 *T*. *pallidum*–positive swab samples, collected over 42 months, from persons with chronic ulcers on Lihir Island; 194 (83.6%) samples could be completely typed.

### Sequence diversity of the selected polymorphic markers

#### tp0548

**Fig 1:**
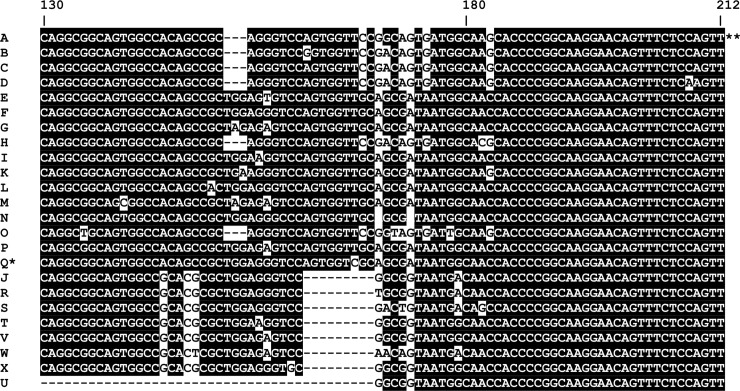
Sequence alignment of *Tp0548* types A through X. Sequence alignment is for different strains of *T*. *pallidum* subsp. *pallidum* and *T*. *pallidum* subsp. *pertenue*. The coordinates of nucleotides 130–212 shown above the alignment are based on the Nichols strain genome (AE 000520.1) as indicated by **. Published reference sequences for each *tp0548* type are as follows: Types A-I:[11]; Type J: [36]; Type K: [17]; Type L: [37]; Type M-N:[38]; Type O:[33]; Type P: [34] Type Q: [35]; Type R,V,W: [5]; Type S-T and X: this work; Type U: [7] Type Q (indicated by *) was originally incorrectly published as Type O; it was renamed in this manuscript.

Sequence analysis of nucleotides 130–212 (cognate to Nichols strain genome [AE 000520.1]) of *tp0548* from the published genomes and newly sequenced *tp0548* loci of historical yaws strains resulted in the identification of four *tp0548* genotypes types (designated here as types R, V, W, and X. Type O had been defined by Knauf *et al*.[33]. While this manuscript was under review, two new *tp0548* types were published: one was defined as P by Mikalova *et al*. [34] and the other was incorrectly defined as type O by Li *et al et al*. [35] (here re-defined as Q). Analysis of the PNG samples resulted in the identification of two *tp0548* genotypes (sequences designated S and T) that had not previously been identified in the literature. The Fribourg-Blanc isolate *tp0548* sequence has a 47 bp deletion from coordinates 122–168 (cognate to the Nichols strain) [7] and was assigned type U (Fig 1).

In our analysis of the PNG samples, we found a large number (n = 160) of samples with the previously described type “J” *tp0548* sequence, first identified in Paris by Grange *et al*. in a genital ulcer of a man with recent sexual exposure in Pakistan [36]. In developing the nomenclature for the *T*.*p*. *pertenue* typing system, we debated whether to continue adding letters to the already extensive list of *tp0548* type sequences (Fig 1) but, when the Paris sample (subsequently determined to be *T*. *pallidum* subsp. *endemicum*) was used to define type “J” in that list, we elected to continue adding the new PNG sequences to the existing list.

**Fig 2:**
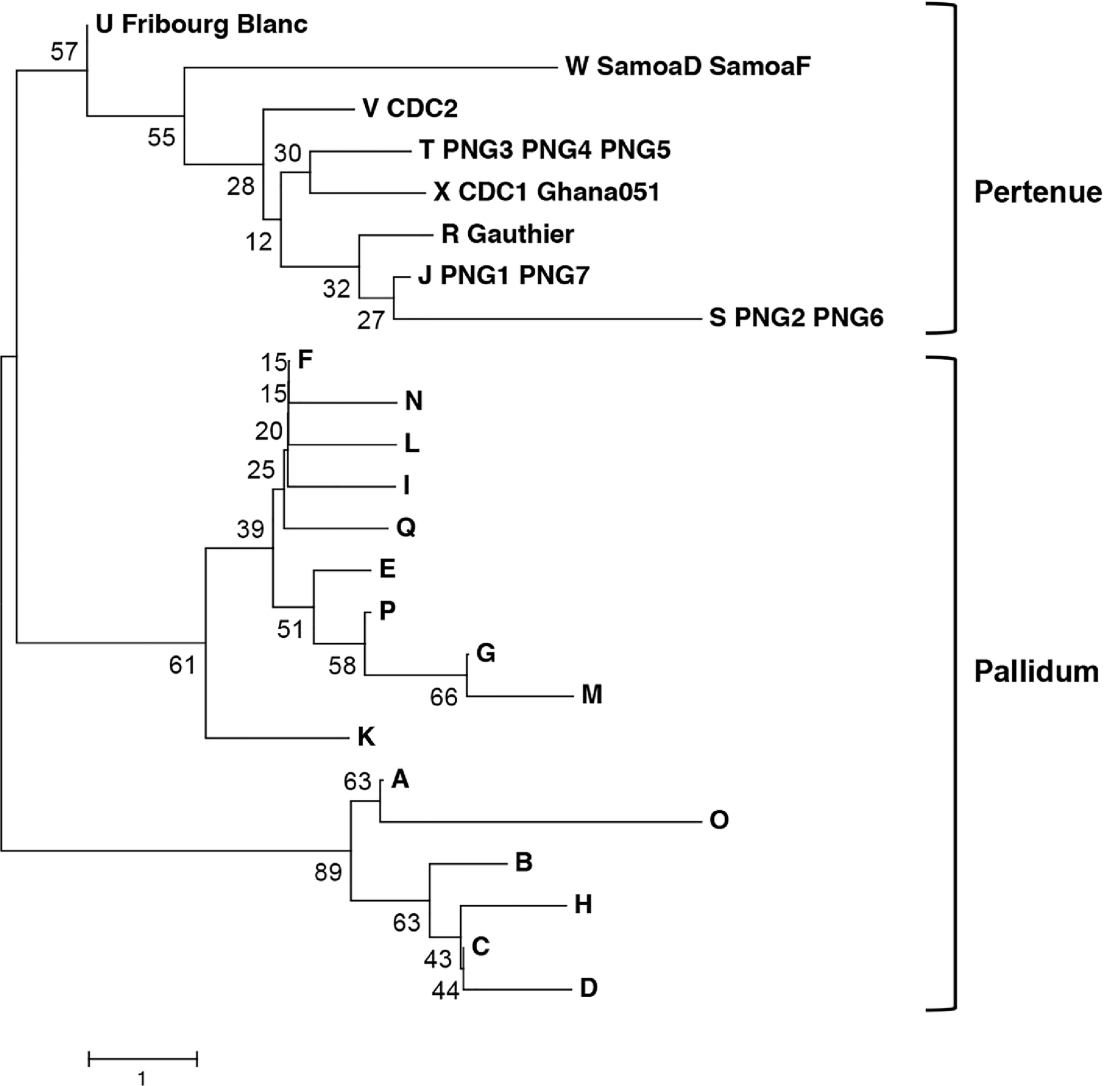
Phylogenetic relationships of the *tp0548* types. *tp0548* types are shown for *T*.*p*. *pallidum*, *T*.*p*. *pertenue*, and Fribourg Blanc isolates/strains and for PNG samples, as shown in Fig 1. Sequences were first aligned using the Muscle algorithm, using default parameters. The evolutionary history was inferred using the Neighbor-Joining method. The optimal tree is shown, with branch lengths equivalent to the evolutionary distance as indicated by the scale. Evolutionary distance was measured using the number of differences per sequence, with pairwise deletion of gaps. The percentage of replicate trees in which the associated molecular types clustered together in the bootstrap test (1000 replicates) is shown next to the branches. Analyses were conducted in MEGA version 7.0 [32].

Phylogenetic analysis of the *tp0548* typing sequences divides the Treponema into three clades, two containing subspecies *pallidum* strains and one containing the *pertenue* strains (Fig 2). Based on *tp0548* alone, there was limited bootstrap support to divide the *pertenue* types, with type W being the most distinct, albeit with a bootstrap value of only 51.

#### tp0136

**Fig 3:**
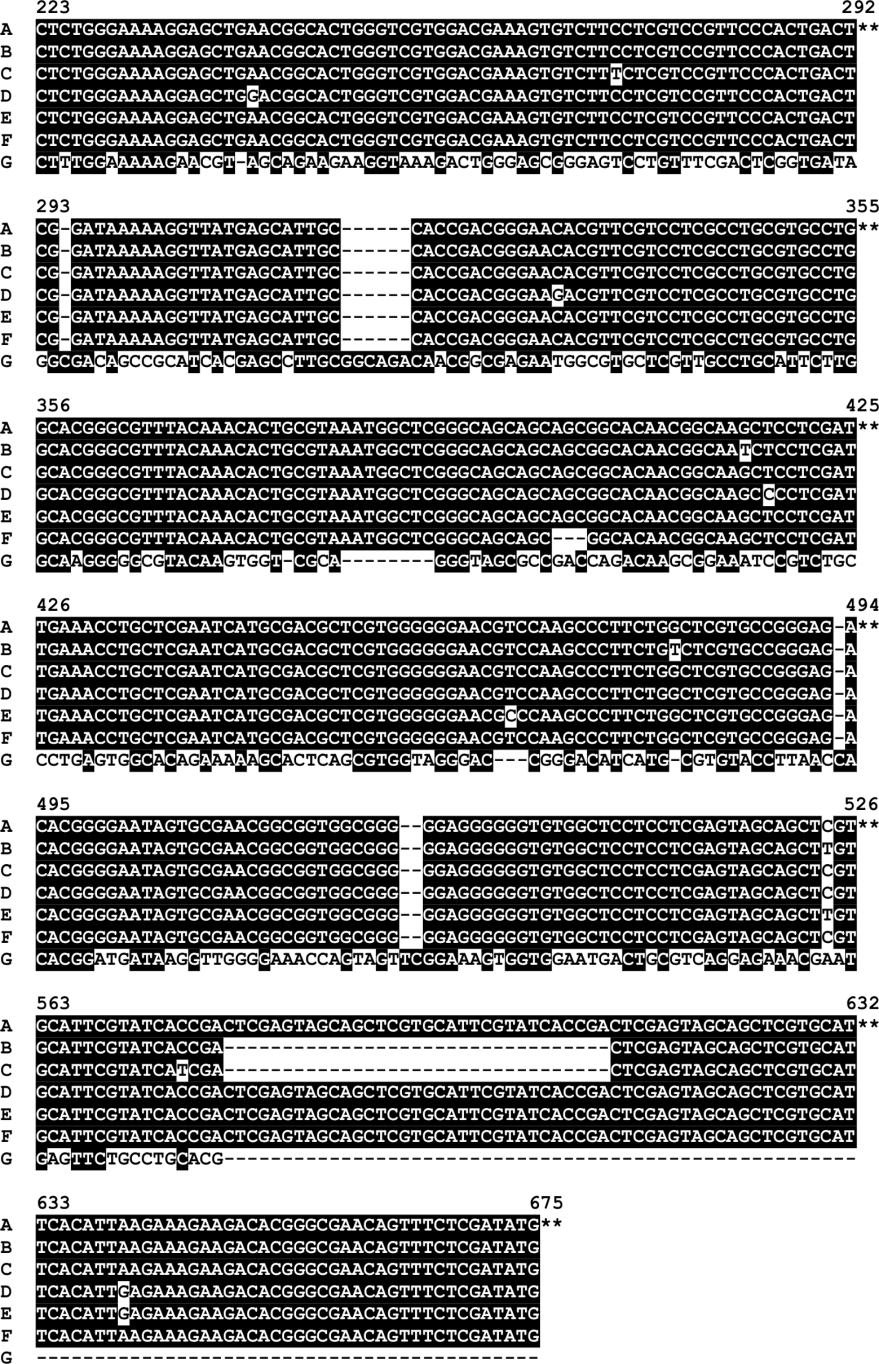
Sequence alignment of *Tp0136* types A through G from *T*. *pallidum* subsp. *pertenue* isolates and Papua New Guinea samples. The coordinates in the alignment between nucleotides 223 and 675 in *T*. *pallidum* subsp. *pertenue* strains and Papua Guinea samples are in reference to strain CDC2 in GenBank (Accession No. CP002375.1) as indicated by **. **A**: Fribourg-Blanc, CDC2; **B**: Samoa D, Samoa F; **C**: Gauthier, **F**: CDC1, Ghana051; **D, E**, and **G**: Papua New Guinea samples.

**Fig 4:**
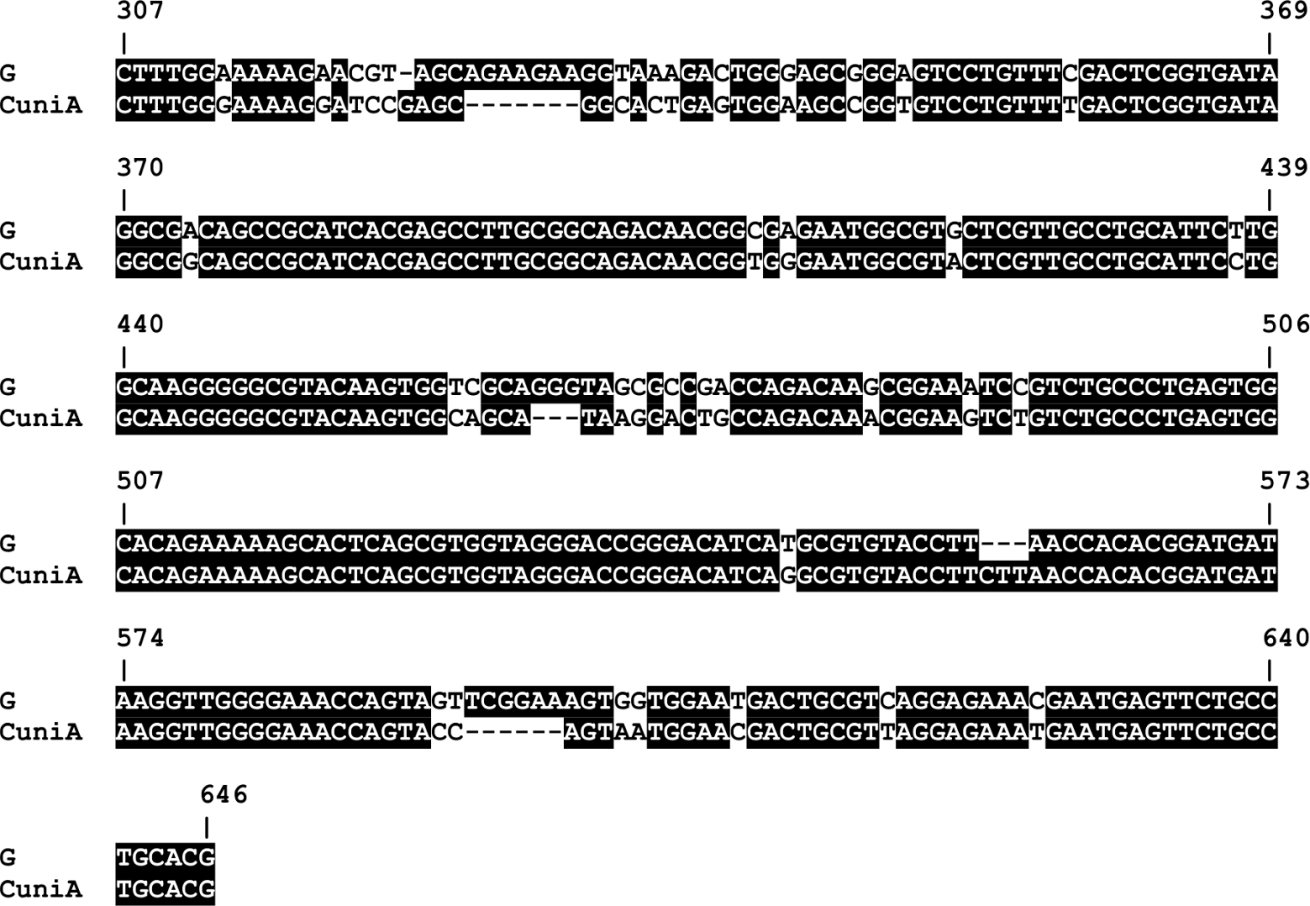
Sequence alignment of *tp0136* type G with *Treponema paraluiscuniculi* A. The very unusual sequence (Type G) found in *tp0136* from the majority of PNG samples was more closely aligned with the sequence from *T*. *paraluiscuniculi* than with the other *T*.*p*. *pertenue* strains (types A-F in Fig 3). The coordinates in the alignment are in reference to *T*. *paraluiscuniculi* A strain (Accession No. CP002103) as indicated by **.

Sequence diversity in *tp0136* has been described by others [8,21]. In addition, Flasarova *et al*. used the *Tp0136* gene as an adjunct to the CDC typing method for *T*.*p*. *pallidum* strains and have found sequence variation among nucleotides 303–1452 [15,16]. Because we wanted to focus on smaller amplicons for greater sensitivity in typing clinical samples, we designed primers that amplified the region between nucleotides 223–675 (cognate to CDC2 sequence). These primers successfully amplified the historical/reference strains (Table 1) and a subset of the PNG clinical samples, resulting in the identification of four types (A-D) in the historical yaws strains and two additional *tp0136* types (E-F) in the PNG samples (Fig 3). A large subset (n = 164, 70.7%) of the PNG samples could not be amplified by these primers and we therefore designed another antisense primer (Antisense 2, Table 3) which successfully amplified the *tp0136* typing region from this latter group of samples. Sequence analysis showed that these samples contained a *tp0136* sequence with relatively high divergence compared to the other sequences, and this was designated type G (Fig 3). Interestingly, BLAST analysis indicated that this sequence was similar to the *tp0136* sequence from *Treponema paraluiscuniculi A* [39] as shown in Fig 4.

**Fig 5:**
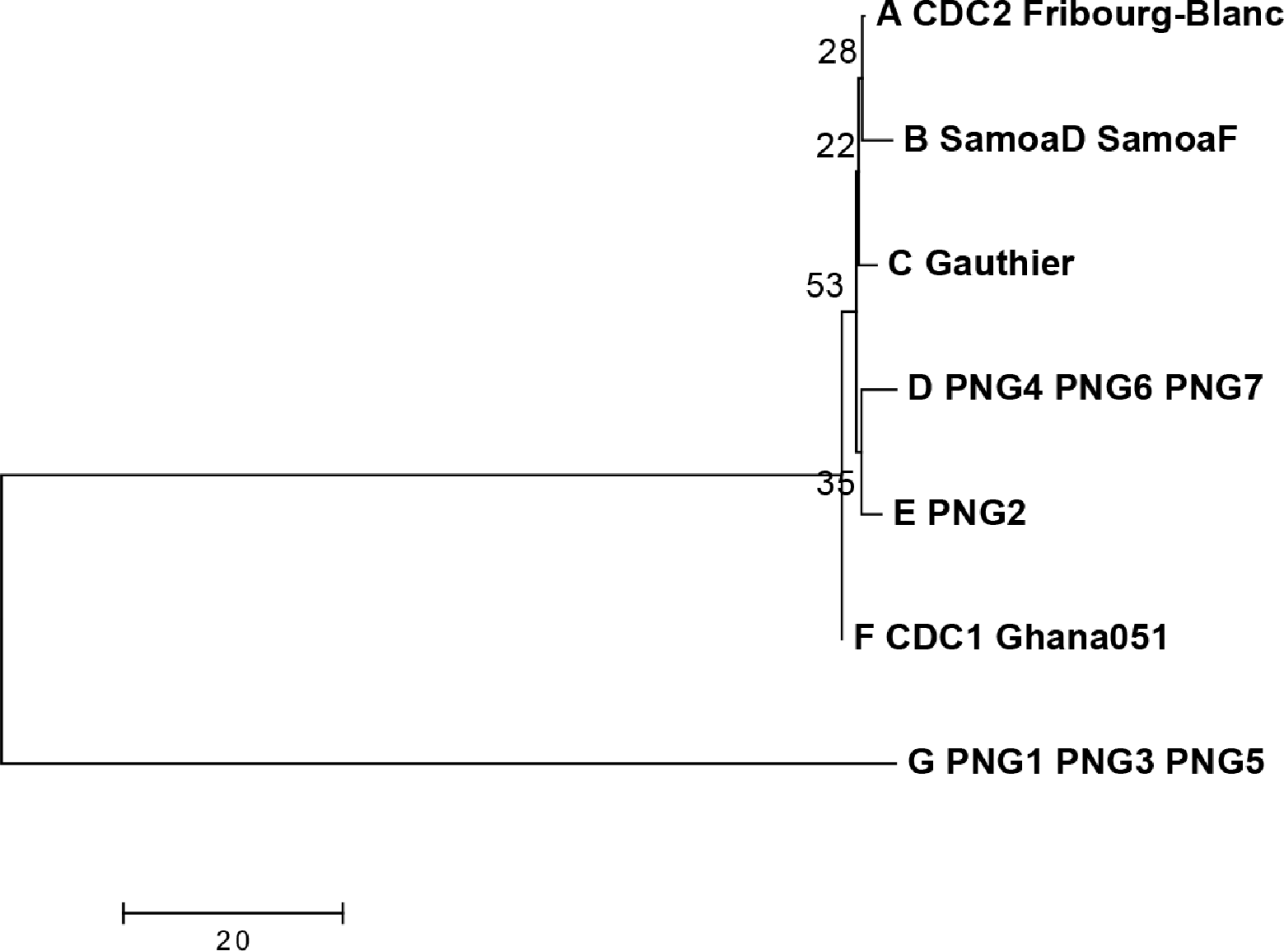
Phylogenetic relationships of the *tp0136* typing region. *tp0136* types are shown for *T*.*p*. *pertenue* and Fribourg Blanc isolates and PNG samples; typing designations are as described in Fig 3. Sequences were first aligned using the Muscle algorithm, using default parameters. The evolutionary history was inferred using the Neighbor-Joining method. The optimal tree is shown, with branch lengths equivalent to the evolutionary distance as indicated by the scale. Evolutionary distance was measured using the number of differences per sequence, with pairwise deletion of gaps. The percentage of replicate trees in which the associated molecular types clustered together in the bootstrap test (1000 replicates) is shown next to the branches. Analyses were conducted in MEGA version 7.0 [32].

In the phylogenetic analysis (Fig 5), the *tp0136* marker divides the PNG strains into two major clusters, with one containing all of the historical strains and the four very closely related PNG2, 4, 6, and 7 types, while type G, found in three groups (PNG1, 3, 5), showed high divergence from all other types as indicated by the multiple alignments.

#### tp0326

**Fig 6:**
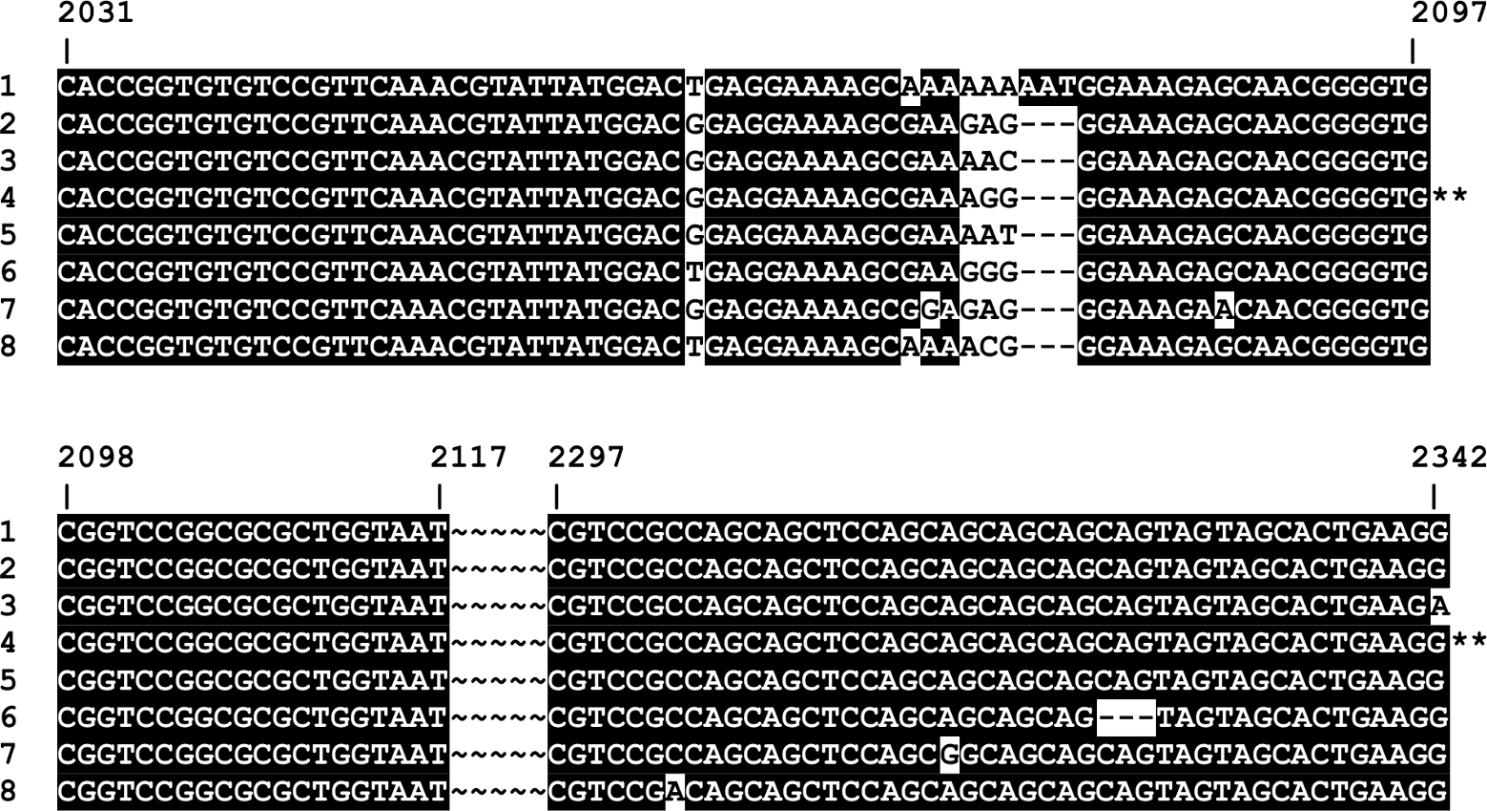
Sequence alignment of *tp0326* typing region. Sequence alignment of *tp0326* types from *T*. *pallidum* subsp. *pertenue* isolates and Papua New Guinea samples. Nucleotide numbering between 2031and 2342 in the alignment refers to coordinates in strain CDC2 in GenBank (Accession No. CP002375.1) as indicated by **. The sequences between coordinates 2117 and 2297 are conserved in all strains examined. 1: Samoa D, Samoa F; 2: Gauthier; 3: Ghana051, CDC1; 4: CDC2; 5: Fribourg-Blanc; 6–8: Papua New Guinea samples.

Sequence diversity in the third typing target, *tp0326* (originally called Tp92), was initially demonstrated by Cameron *et al*. [19]. This gene encodes an orthologue of BamA, which is part of the outer membrane protein assembly machinery [20]. The *tp0326* typing region (nucleotides 2031–2345, cognate to CDC2) defined five genotypes (designated 1–5) among the historical strains and three new genotypes (designated 6–8) in the PNG samples (Fig 6).

**Fig 7:**
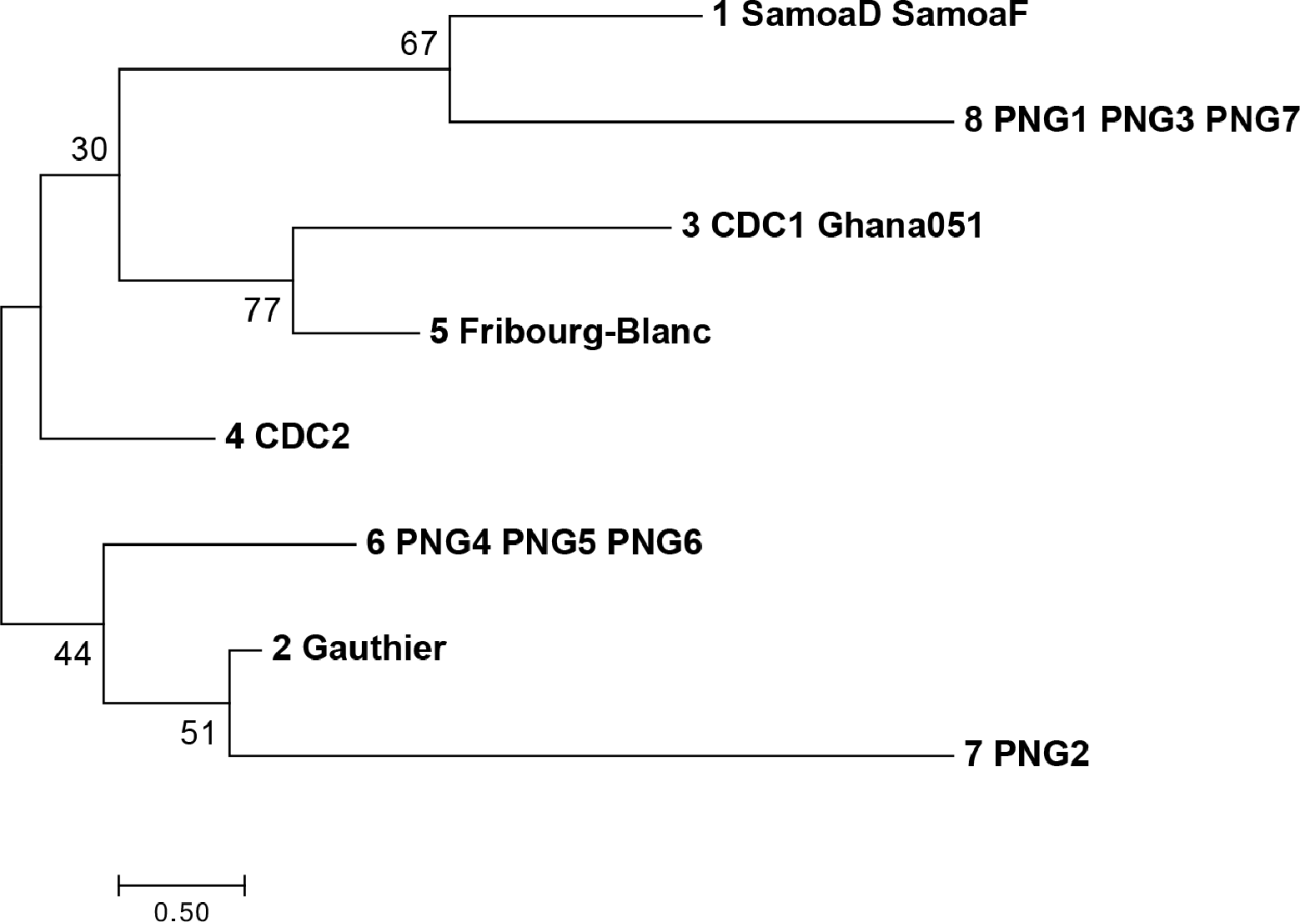
Phylogenetic relationships of the *tp0326* types. *tp0326* types are shown for *T*.*p*. *pertenue* and Fribourg Blanc isolates and PNG samples; typing designations are as described in Fig 6. Sequences were first aligned using the Muscle algorithm, using default parameters. The evolutionary history was inferred using the Neighbor-Joining method. The optimal tree is shown, with branch lengths equivalent to the evolutionary distance as indicated by the scale. Evolutionary distance was measured using the number of differences per sequence, with pairwise deletion of gaps. The percentage of replicate trees in which the associated molecular types clustered together in the bootstrap test (1000 replicates) is shown next to the branches. Analyses were conducted in MEGA version 7.0 [32].

Phylogenetic analysis of *tp0326* showed the relatively low diversity of this marker, however the polymorphisms present divided the eight groups into distinct clusters (Fig 7). Interestingly, PNG groups 2, 4, 5, and 6, clustered with the Gauthier strain, which was isolated in Africa, while PNG groups 1, 3, and 7 clustered with Samoa D and F strains, which were isolated in the South Pacific, near Papua New Guinea.

### MLST typing of *T*.*p*. *pertenue* from a yaws-endemic area of Papua New Guinea

**Fig 8:**
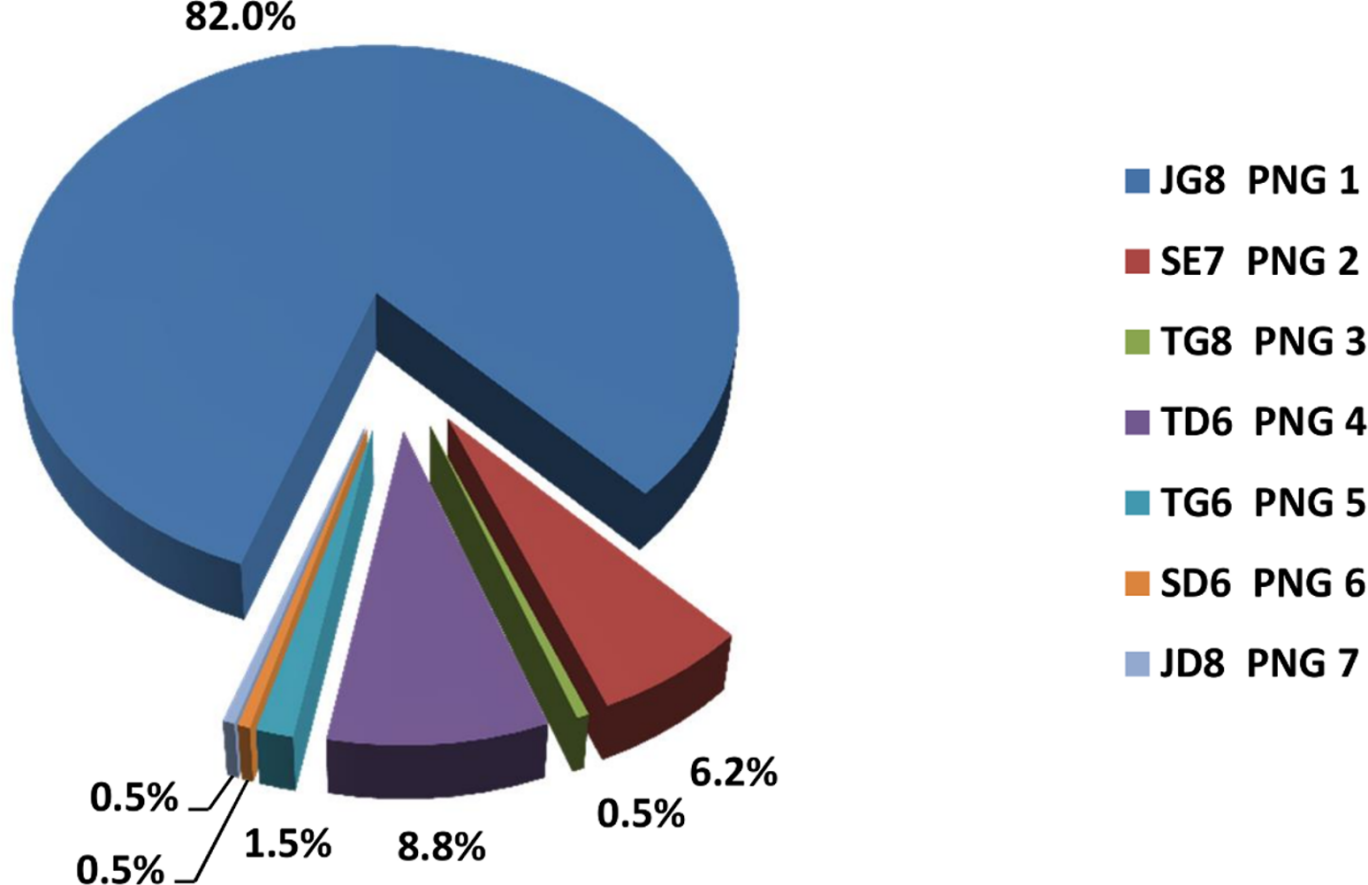
*T*. *pallidum* subsp. *pertenue* types collected on Lihir Island, Papua New Guinea, from May 2013 and October 2016. Data for the 194 fully typeable samples are included here, and proportions for each type are shown.

The MLST typing approach based on the three chosen markers was then applied to *T*.*p*. *pertenue* isolates from PNG. Of the 232 total PNG *T*.*p*. *pertenue*-containing samples, 194 (83.6%) were successfully typed at all three loci; 22 (9%) could be partially typed; and 16 (7%) could not be typed at all. Only fully typed samples were further analyzed. During the 3.5 years of subsequent surveys and sampling, a total of seven types (dividing the samples into groups PNG 1 to PNG 7) were observed, with type JG8 (PNG 1) being predominant throughout that period (82%, Fig 8). The distribution of the molecular types during the course of the survey is discussed elsewhere (manuscript submitted).

**Fig 9:**
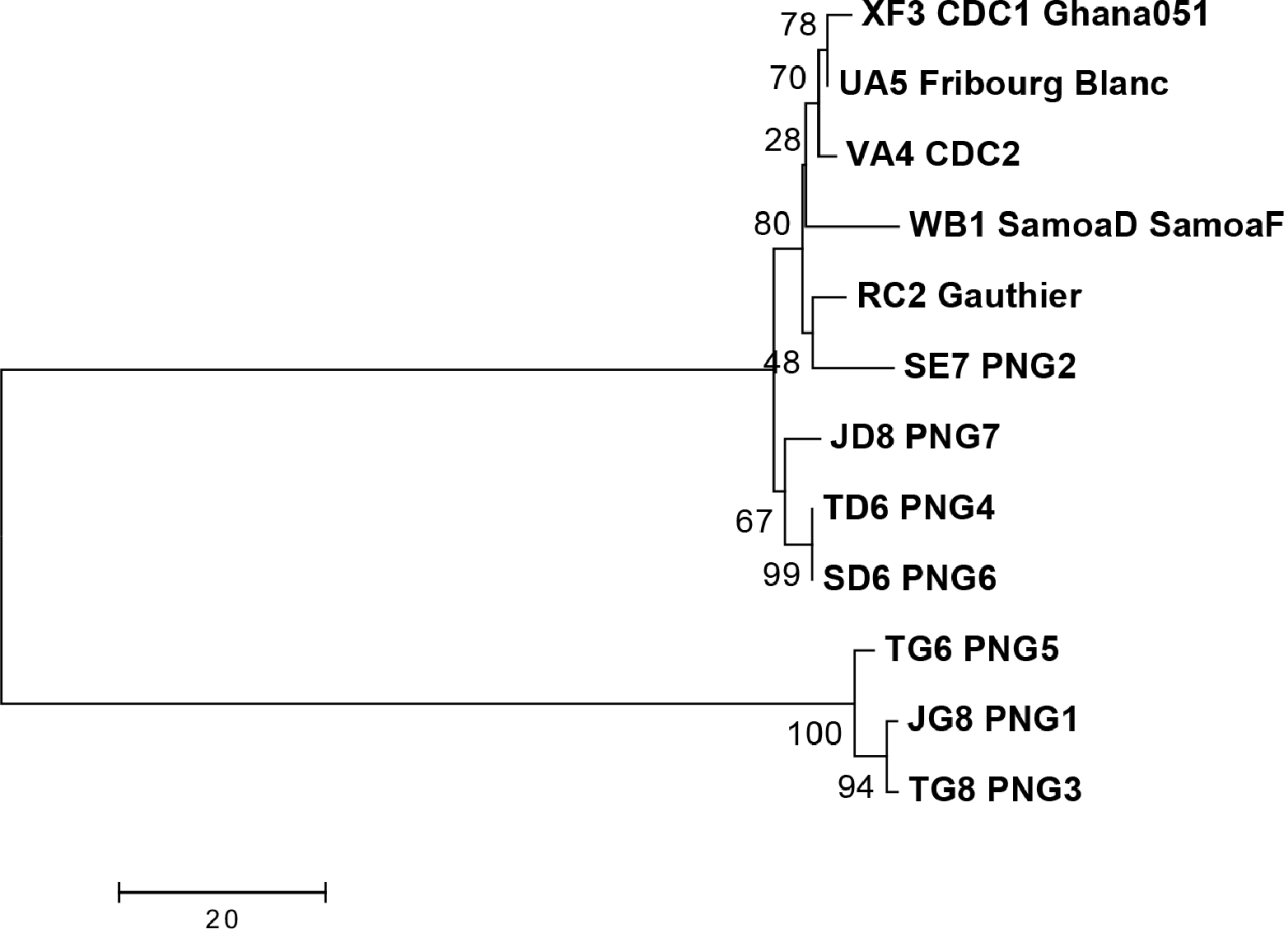
Phylogenetic analysis of *Tp* multilocus sequence types. Multilocus Sequence Types (MLSTs) were defined by sequencing regions of three genes: *tp0548*, *tp0136* and *tp0326*. Concatenated sequences were first aligned using the Muscle algorithm, using default parameters. The evolutionary history of the MLSTs was inferred using the Neighbor-Joining method. The optimal tree is shown, with branch lengths equivalent to the evolutionary distance as indicated by the scale. Evolutionary distance was measured using the number of differences per sequence, with pairwise deletion of gaps. The percentage of replicate trees in which the associated molecular types clustered together in the bootstrap test (1000 replicates) is shown next to the branches. Analyses were conducted in MEGA version 7.0 [32].

A phylogenetic analysis of the final tripartite MLST system for *T*.*p*. *pertenue*, based upon the haplotypes (e.g. concatenated *tp0548*, *tp0136*, and *tp0326* genotypes), is shown in Fig 9. This divided the haplotypes into two major clusters with high bootstrap values, with one containing the three haplotypes with the divergent *tp0136* G genotype (PNG 1,3,5), and the other cluster comprising two minor clusters, one containing all historical isolates and the PNG 2 haplotype (SE7), and the other containing the haplotypes with the *tp0136* D genotype (PNG 4,6,7).

## Discussion

Whole genome sequencing of the Samoa D, Gauthier, and CDC2 *T*.*p*. *pertenue* strains provided an excellent resource for beginning to develop a genotyping tool for yaws clinical samples [4]. For several years, a molecular typing method originally developed at the Centers for Disease Control [10] has been used to identify circulating strains of *T*. *pallidum* subsp. *pallidum* for epidemiological studies [10,11,16,18,40,41]. The enhanced typing method developed by Marra *et al*. built upon the earlier method, proved to provide greater discrimination, and has been widely adopted for typing syphilis strains [11,13,16,41]. Similarly, a typing scheme for yaws organisms could help to inform WHO’s yaws eradication program by permitting an examination of the diversity, stability, and movement of strains throughout a geographical area, and the importation of strains by travelers. The typing system will provide a tool to help to identify the resilience of a bacterial population (e.g. the emergence or importation of strains with enhanced virulence or drug resistance, or the occurrence of an outbreak). Also, the new strain-typing technique will help to improve the understanding of yaws transmission pathways, which will inform the development of improved management and preventative interventions. For example, this tool will help to determine the degree to which yaws cases are clustered within villages and districts; identifying the mechanisms for that clustering could contribute to determination of optimal implementation units for interventions. If inter-village yaws transmission were to be identified, public health officials might want to consider establishing larger implementation units. For evaluating clinical episodes, molecular typing may clarify whether repeated episodes of yaws are due to reinfection rather than relapse in patients in whom genotypically different strains of *T*.*p*. *pertenue* were detected from lesions during each of the separate episodes of ulcer.

Because of the significant difficulty inherent to the syphilis typing method, which relies heavily on analysis of restriction fragment length polymorphisms and of variable numbers of repeats, we sought to develop a multilocus sequence typing (MLST) approach for yaws samples that would be more straightforward and reliable to execute and would provide greater resolution while limiting ambiguous results. Based upon our analysis of sequenced yaws strain genomes and a subset of PNG samples, we chose fragments of the *tp0136*, *tp0548*, and *tp0326* genes as the most promising targets for a *T*.*p*. *pertenue* typing system. Our selection was based primarily upon the level of strain discrimination afforded by the genes and the robustness of the PCR assay in samples containing low concentration of treponemal DNA. We weighed the increased cost and time of adding more targets with the risk of losing the ability to fully type some samples. We fully recognize that, by limiting the size of the gene fragments used in the typing system, we risk losing some discriminating capability. Our experience with typing clinical samples, often from distant locations where optimal handling of DNA is not practical, has convinced us however that the ability to derive a complete molecular typing designation from a high proportion of samples is preferable to a more discriminating system in which a lower percentage of samples can be fully typed. We do not exclude, however, that in the future additional targets might be added to our MLST. Preliminary evidence suggests, for example, that *tp0488* might be a suitable typing targets for *T*. *pallidum* subsp. *pertenue*, and its use should be further evaluated.

Evidence for the utility of our novel *T*.*p*. *pertenue* typing system can be found by examining the strain types of the six historical yaws treponemes, which were collected from disparate geographical regions over nearly 3 decades, and could be divided into four molecular types based on our typing system. It was not unexpected to see that Samoa D and Samoa F, which were both isolated from children in Apia, Western Samoa, in January,1953 [25], had the identical molecular type, WB1. Typing and careful literature research can also lead to questioning of the origins of some DNA samples. We initially conducted typing analysis on DNA from two strains (called CDC2571 and Brazzaville) obtained from a laboratory in the Netherlands, and for which no known isolated strains exist. In carefully researching the origin of this DNA, we were unable to find published references describing the isolation of either strain by those names. In our typing analysis, we found that the Brazzaville strain had identical type sequences to the Gauthier strain (S2 Fig). The 1963 publication describing the isolation of the Gauthier strain [23] describes the collection of a sample from Nigeria in 1960 by a physician in Brazzaville; this publication names the sample “Gauthier, Eastern Nigeria”. We therefore suspect that the “Brazzaville strain” is actually the same as the Gauthier isolate.

Similarly, CDC2571 had the same type sequences as CDC1 and Ghana051 (S3 Fig). There is no known description of the isolation of CDC2575 which was provided to the Netherlands lab by Dr. Peter Perine [42]. The cited reference [24] for CDC2575 describes the isolation in hamsters of treponemes from three children with yaws; all hamster inoculations were conducted on the same date, and the children were residents of two towns in Ghana. Only two of the three strains were successfully transferred and propagated in subsequent animals, and these two are named CDC1 and CDC2; the third un-named strain was apparently lost. We therefore suspect that CDC2575 is actually strain CDC1.

The reference that is typically cited for strain Ghana051 [26] describes the 1988 isolation of the organism from a child who had recently emigrated from Ghana, although this publication does not name the strain. While this manuscript was under review, a publication from Strouhal *et al*. [43] described the genome sequences of CDC2575 and Ghana051, which were virtually identical. The existence of a description of the isolation of the Ghana051 strain and the clear difference in years of reported isolation suggests that Ghana051 (1988) is actually a different strain from CDC1 (1980) and CDC2575 (no description of isolation). The lack of published strain nomenclature for the 1988 isolate leaves the question open, however, as to whether strains were confused or mislabeled during passage or handling over the years. Even whole genome sequencing cannot always determine whether strain mislabeling has occurred.

The utility of strain typing is also apparent in the saga of the Paris case report by Grange *et al*. [36]. The penile lesion was initially thought to be caused by *T*.*p*. *pallidum* acquired by sexual contact in Pakistan, but the *tp0548* sequence, named type J, suggested that it was *T*.*p*. *pertenue*. It was the astute observation of the unusual sequence, called type J, by Mikalova *et al*. [44] that suggested that the agent was not a *pallidum* subspecies. Subsequent more extensive analyses suggest that the treponeme present in this ulcer is actually most closely related to *T*. *pallidum* subsp. *endemicum*, the cause of bejel or endemic syphilis. It has been proposed by Mikalova *et al*. that the *tp0548* sequence from this patient is the result of recombination between *pertenue* and *endemicum* subspecies [45]. Notably, *tp0548* type J is the most prevalent type in the PNG samples that we examined, demonstrating that the *tp0548* type J sequence is seen in modern *T*.*p*. *pertenue* strains, as well as in the putative hybrid *T*.*p*. *endemicum* strain that was presumably sexually acquired in Pakistan. The “Paris” sample also provides evidence that the oft-stated belief that only *T*.*p*. *pallidum* is sexually transmitted is not true. With more molecular analyses being conducted on pathogenic *Treponema*, we increasingly realize that the strict “distinctions” concerning the modes of transmission and, potentially, the clinical manifestations of the *T*.*p*. subspecies are becoming significantly blurred [2].

The overlap among subspecies in transmission and clinical manifestations is further suggested by the finding that the agent causing genital ulcerations (typically ascribed to the *pallidum* subspecies) in wild baboons [46] is most closely related to the yaws-causing *pertenue* subspecies. Subsequent analyses of the material from these animals revealed a *pertenue*-like lineage that was nonetheless distinct compared to the historical human yaws strains [47]. It is striking that analysis of DNA from flies associated with baboon lesions [33] revealed that some flies contained *tp0548* sequences that clustered with the *pertenue* subspecies, while others contained Type J *tp0548* sequences, discussed above as having been first identified in a *T*. *pallidum* subsp. *endemicum* human genital ulcer swab [36,44,45] and later found by us in the majority of samples from children with yaws (molecularly defined as *pertenue*) in Papua New Guinea. Molecular typing and gene sequencing has revealed the intersection of the subspecies [30,45,48].

This picture is further complicated by our finding that a majority of the PNG samples described in this study have a *tp0136* allele that has previously been described only in *Treponema paraluiscuniculi*, which causes a venereal infection in wild rabbits and is thought not to be infectious for humans [49]. In other cases in which alleles thought to belong to one subspecies are found in another subspecies, it has been proposed that inter-subspecies recombination has occurred [45,48]. Might our finding represent an example of possible recombination between two treponemal species?

Aside from triggering deeper evaluations of the nature of *T*. *pallidum* subspecies discussed above, the establishment of a typing system for a pathogen might assist in assessing the association of a particular molecular type with a disease manifestation. If clear associations can be determined through careful epidemiological studies, typing could have a predictive value for regional clinicians and public health officials. For example, if a *T*.*p*. *pertenue* type strain associated with severe joint inflammation were found to be circulating in a community, local health workers could be on heightened alert for identifying and treating such cases. If associations are strong enough, it might justify the adoption of a typing system in routine surveillance programs or in clinical laboratories. Identification of links between genotype and clinical manifestations in yaws is speculative at this time and awaits further study, but a few studies have found associations of specific *T*. *p*. *pallidum* strain types and syphilis manifestations. For example, the 14D/f strain type of *T*.*p*. *pallidum* was significantly associated with neurosyphilis in a large prospective study [11]. In more recent studies, a cluster of *T*.*p*. *pallidum* type 8D/g strains was seen in cases of ocular syphilis in Seattle [12], and infection with the 14I/a type was found to be a significant predictor of serofast status among syphilis-infected patients [50].

With regard to yaws, infection is commonly believed not to affect the cardiovascular and central nervous systems, and not to be transmitted to the fetus during pregnancy. This oft-repeated “maxim” may reflect lack of extensive knowledge on the pathogenesis of yaws. Alternatively, there may be differences in strain invasiveness. Studies conducted by Edington identified syphilis-like aortitis as a major cause of death in people from Ghana where yaws is endemic [51], while Roman and Roman suggested that there is evidence in the literature to support not only neurological and cardiovascular involvement in yaws patients, but also vertical transmission of the pathogen [52]. In the future, discordant observations and conclusions concerning yaws pathogenesis and manifestations may be explained by genetic differences among strains, and with sufficient clinical data, our typing system might assist in linking genotype and phenotype in *T*.*p*. *pertenue*.

In summary, we have described a new sequence-based typing system for *T*. *pallidum* subsp. *pertenue*, based upon *tp0548*, *tp0136*, and *tp0326 genes*. The proposed method was developed to maximize the discriminating capability of the sequence target regions, balanced by the robustness of the PCR to amplify samples with limiting amounts of treponemal DNA. In this study, we limited our analysis to the aggregated typing results from clinical samples obtained during the 3.5 years of examinations of the population of Lihir Island. An analysis of the geographical clustering of the strain types across the island and the correlation of strain type with population migration or travel will provide critical information for developing protocols and monitoring progress of yaws eradication activities in the future. Those analyses are ongoing.

While this new typing system has been quite useful in examining strains circulating on Lihir Island, it is very important to assess its applicability to samples from yaws lesions from other geographical regions. It is fully expected that more strain types will be identified as the typing method is applied to more yaws-affected populations, and that modifications to the primer sets may be needed. It should also be remembered that no typing system will be universally sensitive, particularly for samples that cannot be collected, stored, or transported under optimal conditions. The discriminating ability of the typing system described here for historical *T*.*p*. *pertenue* isolates from Pacific Islands and Africa, as well as clinical samples, suggests however that it is a good prototype that will be readily applicable to the current WHO campaign to eliminate yaws.

## Supporting information

S1 Fig Alignment of *tp0619* sequences. Alignment of the *tp0619* sequences from historical strains and the five molecular types identified from 95 PNG samples. The Samoa D *tp0619* sequence is identical to that of all other historical *pertenue* strains analyzed (Samoa F, Gauthier, Brazzaville, CDC 1, CDC 2, CDC2575, and Ghana051).
(TIF)

S2 Fig Alignment of concatenated typing regions of *tp0548* (red), *tp0136* (blue) and *tp0326* (green) from the Gauthier and Brazzaville strains shows identical sequences. (TIF)

S3 Fig Alignment of concatenated typing regions of *tp0548* (red), *tp0136* (blue) and *tp0326* (green) from the CDC1, Ghana051, and CDC2571 strains shows identical sequences. (TIF)
