# Supplementary figures and images for: Development of a Multilocus Sequence Typing (MLST) scheme for Treponema pallidum subsp. pertenue: Application to yaws in Lihir Island, Papua New Guinea
Source: PLoS Negl Trop Dis. 2017 Dec 27;11(12):e0006113. doi: 10.1371/journal.pntd.0006113 (PMC5760108; doi:10.1371/journal.pntd.0006113)

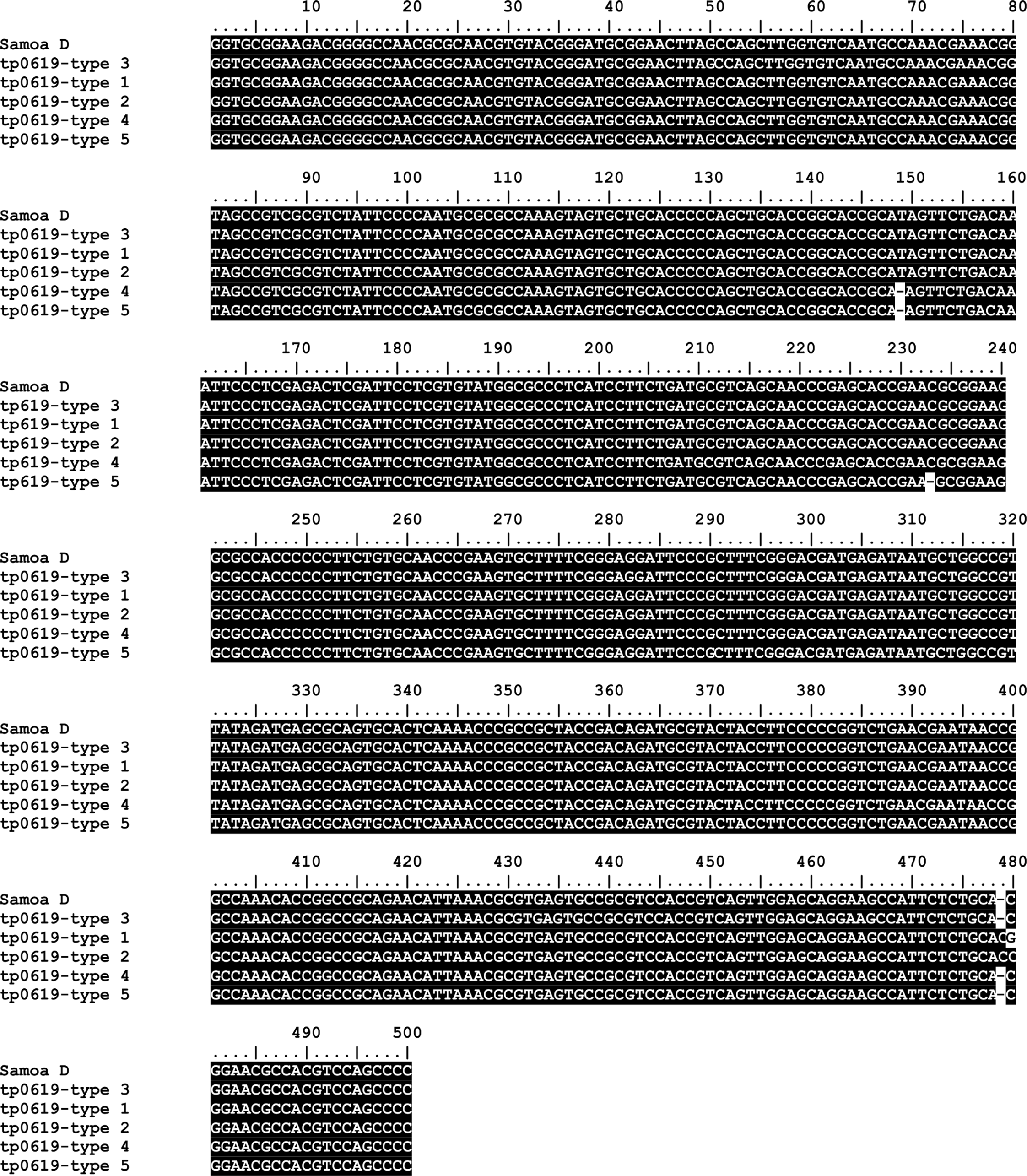

Supplement: S1 Fig — Alignment of the tp0619 sequences from historical strains and the five molecular types identified from 95 PNG samples. The Samoa D tp0619 sequence is identical to that of all other historical pertenue strains analyzed (Samoa F, Gauthier, Brazzaville, CDC 1, CDC 2, CDC2575, and Ghana051). (TIF) [file pntd.0006113.s001.tif]

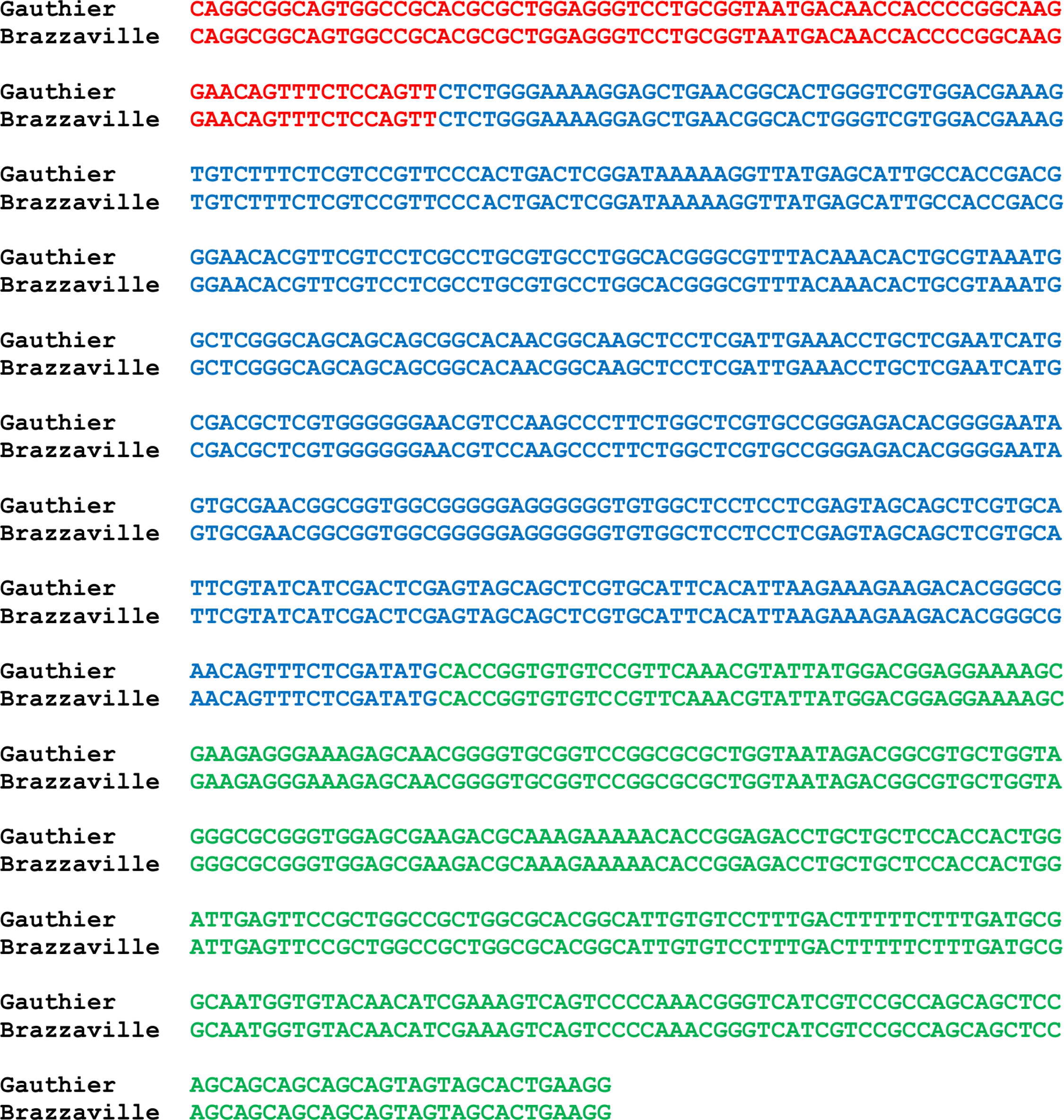

Supplement: S2 Fig — (TIF) [file pntd.0006113.s002.tif]

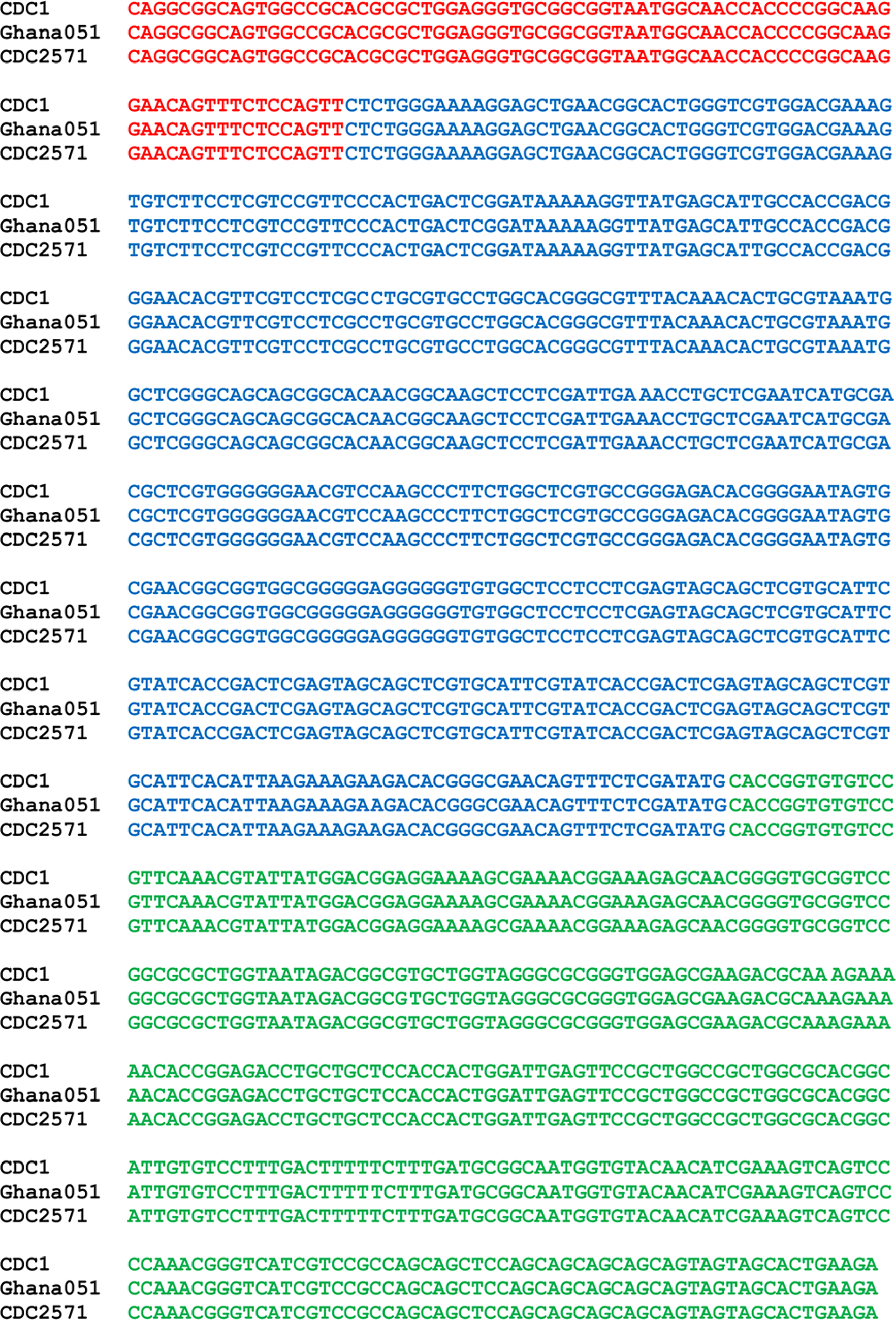

Supplement: S3 Fig — (TIF) [file pntd.0006113.s003.tif]
